# HIV and Mature Dendritic Cells: Trojan Exosomes Riding the Trojan Horse?

**DOI:** 10.1371/journal.ppat.1000740

**Published:** 2010-03-26

**Authors:** Nuria Izquierdo-Useros, Mar Naranjo-Gómez, Itziar Erkizia, Maria Carmen Puertas, Francesc E. Borràs, Julià Blanco, Javier Martinez-Picado

**Affiliations:** 1 IrsiCaixa Foundation, Badalona, Spain; 2 Laboratory of Immunobiology for Research and Application to Diagnosis (LIRAD), Blood and Tissue Bank, Badalona, Spain; 3 Institut d'Investigació en Ciències de la Salut Germans Trias i Pujol, Universitat Autònoma de Barcelona, Badalona, Spain; 4 Institució Catalana de Recerca i Estudis Avançats (ICREA), Barcelona, Spain; University of California San Diego, United States of America

## Abstract

Exosomes are secreted cellular vesicles that can induce specific CD4^+^ T cell responses in vivo when they interact with competent antigen-presenting cells like mature dendritic cells (mDCs). The Trojan exosome hypothesis proposes that retroviruses can take advantage of the cell-encoded intercellular vesicle traffic and exosome exchange pathway, moving between cells in the absence of fusion events in search of adequate target cells. Here, we discuss recent data supporting this hypothesis, which further explains how DCs can capture and internalize retroviruses like HIV-1 in the absence of fusion events, leading to the productive infection of interacting CD4^+^ T cells and contributing to viral spread through a mechanism known as *trans*-infection. We suggest that HIV-1 can exploit an exosome antigen-dissemination pathway intrinsic to mDCs, allowing viral internalization and final *trans*-infection of CD4^+^ T cells. In contrast to previous reports that focus on the ability of immature DCs to capture HIV in the mucosa, this review emphasizes the outstanding role that mature DCs could have promoting *trans*-infection in the lymph node, underscoring a new potential viral dissemination pathway.

## Introduction

Dendritic cells (DCs) scattered throughout the peripheral tissues act like sentinels and recognize a wide range of microorganisms. At this stage, DCs display an immature phenotype. When pathogen invasion takes place, immature DCs (iDCs) can capture microorganisms via endocytic surveillance receptors, resulting in the classical intracellular lytic pathway that permits processing of antigenic peptides [1]. The signaling through receptors or the detection of proinflammatory cytokines prompts iDC activation and migration from the periphery towards the secondary lymphoid organs. Concurrently, co-stimulatory molecules are expressed in the cell membrane, preparing DCs for competent T cell priming. In the T cell areas of the lymph nodes, fully mature DCs (mDCs) present pathogen-derived epitopes to CD4^+^ T or CD8^+^ T lymphocytes. This way, DCs orchestrate immune responses to invading pathogens and have a pivotal role during infections [2].

However, viruses, including the human immunodeficiency virus (HIV), have evolved different strategies to evade DC antiviral activity. Indeed, it has been known for years that DCs exposed to HIV-1 transmit a vigorous cytopathic infection to CD4^+^ T cells [3]. Although the frequency of HIV-1-infected DCs is often 10- to 100-fold lower than that of CD4^+^ T cells [4], DCs do not need to be productively infected to transmit the virus and spread it in an infectious form [5], which is in contrast to other HIV-1 target cells such as CD4^+^ T cells or macrophages. Notably, separate pathways mediate the productive infection of DCs and their ability to capture and internalize HIV-1 in the absence of viral fusion [6]. The latter mechanism involves binding and uptake of HIV-1, traffic of internalized virus, and its final release, allowing transfer to CD4^+^ T cells, a process known as *trans-*infection [5],[7].

*Trans*-infection has been related to the ability of C-type lectin receptors like DC-SIGN expressed in certain DCs to tightly bind to the HIV-1 surface envelope glycoprotein gp120 [8] and endocytose viral particles [9]. The initial identification of DC-SIGN as an HIV receptor permitting *trans*-infection of T cells led to the “Trojan horse” hypothesis, which relates the preliminary establishment of HIV-1 infection to the ability of iDCs to capture the virus via DC-SIGN in the peripheral tissue and then migrate to the lymph nodes, where HIV-1 transferred to CD4^+^ T cells could easily start the spread of infection [5],[7],[10].

**Figure 1:**
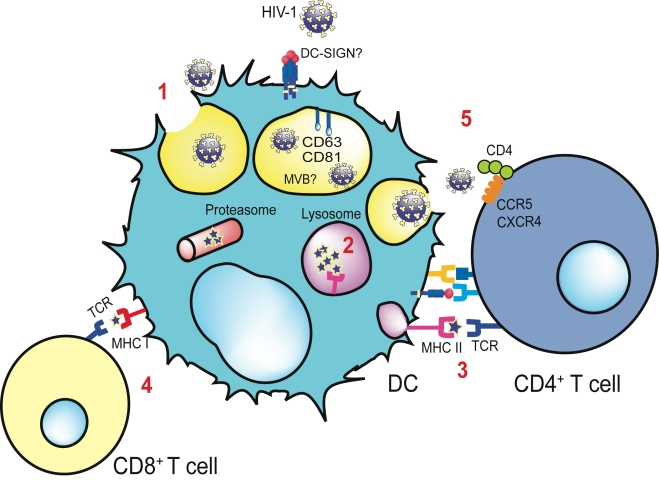
Antigen presentation and *trans*-infection of a CD4^+^ T cell mediated by a DC. Viral binding to distinct cellular receptors allows viral endocytosis via a non-fusogenic mechanism (**1**). The virus is retained in the multivesicular body compartment (MVB), where it is enriched in tetraspanins such as CD81 and CD63. Part of the virus is degraded in the lysosomes (**2**), and viral antigens are presented via MHC-II to the T cell receptor (TCR) of CD4^+^ T cells (**3**) through the formation of an immunological synapse. If an endocytosed virus gains access to the cytoplasm of the DC, it can be processed by the proteasome and crosspresented via MHC-I to CD8^+^ T cells (**4**). Viral transmission takes place when part of the virus evades classical degradation pathways. MVB recycles back and fuses with the plasma membrane, allowing the liberation of entrapped virus and the productive infection of DC-interacting CD4^+^ T cells (**5**), a mechanism known as *trans*-infection. The contact area between an uninfected DC bearing HIV infectious particles and a CD4^+^ T cell is termed the infectious synapse.

Knowing the antigen-presenting capabilities of DCs, one would expect that after HIV interaction with surveillance receptors like DC-SIGN, endocytosed virus would end up in classical lysosomic pathways (Figure 1), where viral antigens are degraded and presented in MHC-II molecules to CD4^+^ T cells [11],[12]. Furthermore, part of the internalized virus could also gain access to the cytoplasm and be processed throughout the proteasome, finally being crosspresented in MHC-I molecules to CD8^+^ T cells [13],[14]. However, in the specific case of HIV interaction with iDCs, it has been proposed that part of the internalized virus escapes these degradation routes and is maintained in endosomal acidic compartments, retaining viral infectivity for the long periods required to promote efficient HIV-1 transfer to CD4^+^ T cells [5],[9].

Despite this preliminary model of viral retention, recent studies have demonstrated that iDCs show rapid degradation of captured viral particles, which do not last more than 24 hours before being processed [14]–[17]. These studies suggest a two-phase mechanism of viral transmission mediated by iDCs: one restricted to a short period through the *trans*-infection process, and a later one due to a long-term transfer of de novo viral particles produced after iDC infection [15],[16],[18].

## Trojan Horses and HIV Transmission: Mature DCs Win the Race

Several results ([17], [19]–[21], reviewed in [22]), indicate that iDCs have reduced *trans*-infection ability. Conversely, mDCs are much less vulnerable to viral fusion events and productive HIV infection than iDCs [23],[24], while displaying a greater ability to capture incoming virions [17],[21],[25], retain them in an infectious form, and transmit them to target T cells through *trans*-infection [17], [19]–[21]. The location of internalized virions is dramatically different in immature and mature DCs [25]. Strikingly, the poorly macropinocytic mDCs [2],[26],[27] sequester significantly more whole, structurally intact virions into large vesicles within the cells, whereas the endocytically active iDCs not only retain fewer internalized virions, but also locate them closer to the cell periphery [25]. This internalization view has been previously challenged, suggesting that cell-surface-bound HIV is the predominant pathway for viral transmission mediated by DCs [28]. However, a recent report on this topic reconciles these two models by demonstrating that HIV resides in an invaginated domain within DCs that is both contiguous with the plasma membrane and distinct from classical endocytic vesicles [29].

**Figure 2:**
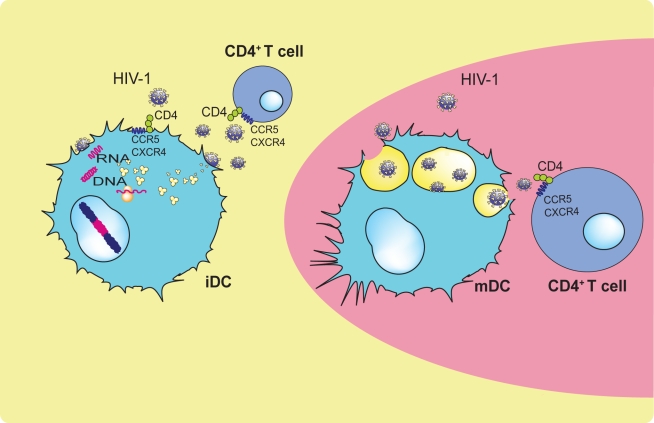
Proposed major roles of iDCs and mDCs during HIV disease progression. Productive infection of iDCs allows viral transmission in the peripheral tissues, while mDC viral capture leads to *trans*-infection in the lymphoid tissues.

Collectively, these results favor a model in which both direct infection and *trans*-infection abilities coexist to a different extent in immature and mature DC subsets. Maturation of DCs enhances viral capture activity and *trans*-infection capacity while diminishing viral fusion events [24] and productive infection [23]. Under these circumstances, iDCs would preferentially transmit de novo synthesized virus upon productive infection [15], and the mDC-enhanced *trans*-infection ability would play a key role in the lymph nodes, mediating viral transmission to new target CD4^+^ T cells (Figure 2).

Given the unique capability of mDCs to promote HIV-1 infection of CD4^+^ T cells in vitro, we hypothesize that in vivo, mDC *trans*-infection could augment viral dissemination in the lymphoid tissue and significantly contribute to HIV disease progression. mDCs have a greater ability to stimulate CD4^+^ T cell proliferation than iDCs [30],[31]. Accordingly, mDCs presenting viral antigens could activate HIV-specific naïve CD4^+^ T cells in the course of their first encounters in the lymph node. As a result, HIV-specific naïve CD4^+^ T cells would undergo several rounds of division during their initial expansion and differentiation into effector CD4^+^ T cells, becoming highly susceptible to actual HIV infection, as has been previously demonstrated [32]. Notably, the viral dissemination that mDCs can potentially mediate in vivo is enormous: T cells approach mDCs randomly and make exploratory contacts that last only minutes, enabling DCs to contact many T cells per hour [33]. Thus, since viral transmission through *trans*-infection does not rely on antigen presentation, many CD4^+^ T cells could be exposed to mDC virus; however, only after antigen presentation would naïve CD4^+^ T cells be activated and their subsequent proliferation render these cells more susceptible to HIV infection.

Once infected, these activated CD4^+^ T cells are known to have short half-lives in vivo, lasting fewer than two days [34]. Therefore, under rapid T cell turnover, DCs could be indispensable to permitting continuous infection of new CD4^+^ T cells [35]. Recently, it has also been suggested that simultaneous priming and infection of T cells by DCs is the main driving force behind the early infection dynamics, when activated CD4^+^ T cell numbers are low [36].

In vivo, the contribution of mDCs to HIV spread might be also supported by the levels of circulating lipopolysaccharide (LPS), which are significantly augmented in chronically HIV-infected individuals, due to the increased translocation of bacteria from the intestinal lumen early after primo infection [37]. The bacterial components released could stimulate DCs systemically, contributing to their maturation and therefore enhancing viral spread while creating the pro-inflammatory milieu associated with chronic HIV infection. This hypothesis is further supported by another report showing that in individuals with HIV-1 viremia, DCs from blood have increased expression levels of co-stimulatory molecules (the hallmark of maturation status) that only diminish when highly active antiretroviral therapy suppresses the viral load [38]. Although the plasma LPS concentration found in HIV^+^ patients is lower than the one used to mature DCs in vitro [17], [19]–[21],[39], it is conceivable that in vivo, higher amounts of LPS could accumulate in the most compartmentalized areas of the mucosa or in the adjacent tissues. Therefore, future experiments should address whether the physiological amounts of LPS found in tissues can trigger the same DC maturation status and viral transmission efficacy described in different reports [17], [19]–[21],[39].

Prior infection with other sexually transmitted pathogens is strongly associated with the sexual transmission of HIV [40]. This implies that the probability of a person acquiring HIV infection is increased when there is a preexisting infection or inflammation of the genital epithelium. Under these circumstances, it is quite likely that mucosal inflammation arising from other sexually transmitted pathogens could directly activate and mature DCs in vivo, promoting HIV settlement and favoring the subsequent spread of the viral infection. Interestingly, a recent report shows that in vitro–activated CD34-derived Langerhans cells mediate the *trans*-infection of HIV [39], suggesting a potential role for these mature cells during the establishment of HIV infection.

Unfortunately, recent failures in HIV prophylactic vaccine trials provide additional corroboration of the prominent role mDCs could be playing during HIV infection in vivo. The STEP HIV vaccine trial evaluated a replication-defective adenovirus type 5 (Ad5) vector, which is a weakened form of a common cold virus, modified to carry HIV genes into the body to induce HIV-specific immune responses. This clinical trial was recently stopped due to the vaccine's lack of efficacy and the 2-fold increase in the incidence of HIV acquisition among vaccinated recipients with increased Ad5-neutralizing antibody titers compared with placebo recipients [41]. Of note, a recent report demonstrates that the Ad5 vector, with its neutralizing antiserum (present in people with prior immunity), induced a more marked DC maturation than the vector alone, as indicated by increased CD86 expression levels, decreased endocytosis, and production of tumor necrosis factor and type I interferons [42]. Furthermore, when the Ad5 vector and the neutralizing antiserum were added to DCs pulsed with HIV, significantly enhanced viral infection was observed in DC–T cell co-cultures compared to controls lacking the neutralizing antiserum. That is why these authors postulate that mDCs from people with prior immunity to Ad5 virus might have activated CD4^+^ T cells in vivo, augmenting their susceptibility to HIV infection [42].

Overall, these results highlight the functional relevance that DC maturation could possess under physiological settings, providing the basis for a chronic permissive environment for HIV-1 infection.

## Maturation Also Enhances Presentation Skills

Why would mDCs accumulate viral particles instead of degrade them? This paradoxical retention mechanism could in fact aid immunological surveillance, allowing mDCs to have a source of antigen to present to T cells in the absence of surrounding virus, sustaining immune responses for prolonged periods. Intriguingly enough, DCs have an inherent mechanism to control endosomal acidification to preserve antigen cross-presentation over time [43]. We hypothesize that HIV-1 could be exploiting this preexisting cellular pathway of antigen uptake and retention inherent to mDCs, favoring and enhancing viral *trans*-infection of CD4^+^ T cells. If this is indeed the case, mDC viral uptake would not rely on the recognition of specific viral proteins, but depend on more ubiquitous signals.

Interestingly, we have recently identified an HIV gp120-independent mechanism of viral binding and endocytosis that is upregulated upon DC maturation [17], further supporting distinct works that have demonstrated that DC-SIGN is not responsible for HIV-1 binding to all DC subsets [21], [44]–[53], and clearly highlighting that additional HIV-1 binding molecules remain to be identified.

Furthermore, several lines of evidence suggest that viral envelope–independent capture of HIV by DCs can allow antigen presentation and induce cytotoxic and humoral immune responses. It has been previously shown that DCs can endocytose viral-like particles (VLPs) and induce immune responses through an endosome-to-cytosol cross-presentation pathway [54]. These VLPs do not have the envelope glycoprotein, meaning that the uptake mechanism could be the same as the one we have shown for virus lacking the envelope glycoprotein [17]. In iDCs, HIV envelope and DC-SIGN-dispensable pathways account for about 50% of the antigen presentation through MHC-II molecules [12]. DCs are also able to capture envelope-pseudotyped HIV Gag VLPs through a DC-SIGN-independent pathway, activating autologous naïve CD4^+^ T cells that are then able to induce primary and secondary responses in an ex vivo immunization assay [55]. Overall, these findings reinforce the idea that envelope-independent capture pathways allow viral antigen presentation, thus favoring immune responses.

## The Role of Exosomes during Antigen Presentation

Although the current view of DC functionality has iDCs encountering an antigen in the periphery and carrying it to lymphoid organs, DCs migrating from the periphery may not always be the ones that present the captured antigen in the lymph nodes. Rather, migrating DCs may transfer their captured antigens to other DCs for presentation. The transfer could occur either by the phagocytosis of antigen-loaded DC fragments by another DC [56] or by the release of antigen-bearing vesicles termed exosomes [57]. During periods of pathogen invasion, these exosomes could act as real couriers, increasing the number of DCs bearing a particular epitope, thus amplifying the initiation of primary adaptive immune responses [58].

Interestingly, as it happens with viral particles, exosomes are also internalized and stored in endocytic compartments by DCs, a prerequisite needed to induce different immune responses. Notably, exosomes do not induce naïve T cell proliferation in vitro unless mDCs are also present, indicating that exosomes do not overcome the need for a competent antigen-presenting cell to stimulate T cells. Exosomes from cultured DCs loaded with tumor-derived epitopes on MHC-I molecules are able to stimulate cytotoxic T lymphocyte–mediated anti-tumor responses in vivo [59]. Moreover, it has been demonstrated that tumor cells secrete exosomes carrying tumor antigens, which, after transfer to DCs, also mediate CD8^+^ T cell–dependent anti-tumor effects [60]. Therefore, distinct studies have shown that exosomes carrying tumor epitopes provide a source of antigen for cross-presentation by DCs.

In addition, exosomes are also able to stimulate antigen-specific naïve CD4^+^ T cell responses in vivo [58],[61]. This stimulation can take place either by reprocessing the antigens contained in the captured exosomes or by the direct presentation of previously processed functional epitope–MHC complexes exposed in the exosome surface [58],[61]. These alternative pathways were characterized when it was observed that mDC populations could be devoid of MHC-II molecules and still stimulate CD4^+^ T cells, because MHC-II molecules were already present on the exosomes [61].

**Figure 3:**
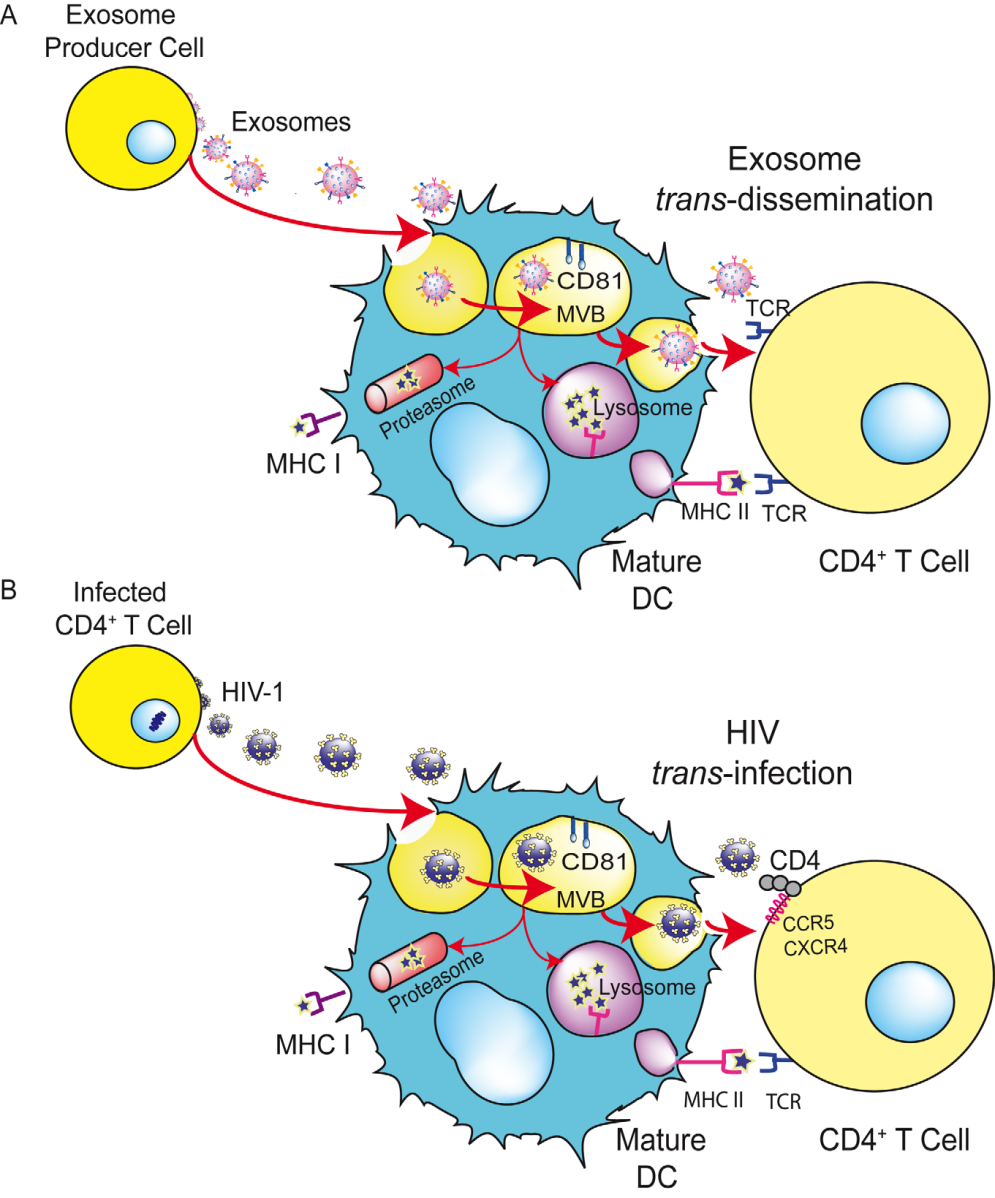
HIV can exploit a preexisting exosome *trans*-dissemination pathway intrinsic to mDCs, allowing the final *trans*-infection of CD4^+^ T cells. (**A**) Exosomes can transfer antigens from infected, tumoral, or antigen-presenting cells to mDCs, increasing the number of DCs bearing a particular antigen and amplifying the initiation of primary adaptive immune responses through the MHC-II pathway, cross-presentation, or the release of intact exosomes, a mechanism described here as *trans-*dissemination. (**B**) HIV gains access into mDCs by hijacking this exosome *trans*-dissemination pathway, thus allowing the final *trans*-infection of CD4^+^ T cells. Adapted from [63] © The American Society of Hematology.

In summary, distinct studies have shown that exosomes can be internalized in DCs, allowing final antigen presentation in the absence of lytic degradation. We suggest that HIV and other retroviruses could be exploiting this exosome antigen dissemination pathway intrinsic to mDCs, allowing the final *trans*-infection of CD4^+^ T cells (Figure 3). In particular, HIV could be hijacking a pathway that exosomes produced by antigen-presenting cells can follow upon capture by mDCs, mediating the indirect activation of CD4^+^ T cells by presenting functional epitope–MHC-II complexes through a *trans*-dissemination mechanism [58],[61]. Our data supports the Trojan exosome hypothesis that proposes that retroviruses take advantage of a cell-encoded intercellular vesicle traffic and exosome exchange pathway, moving between cells in the absence of fusion events [62],[63].

## Are Trojan Exosomes Riding the Trojan Horse?

**Figure 4:**
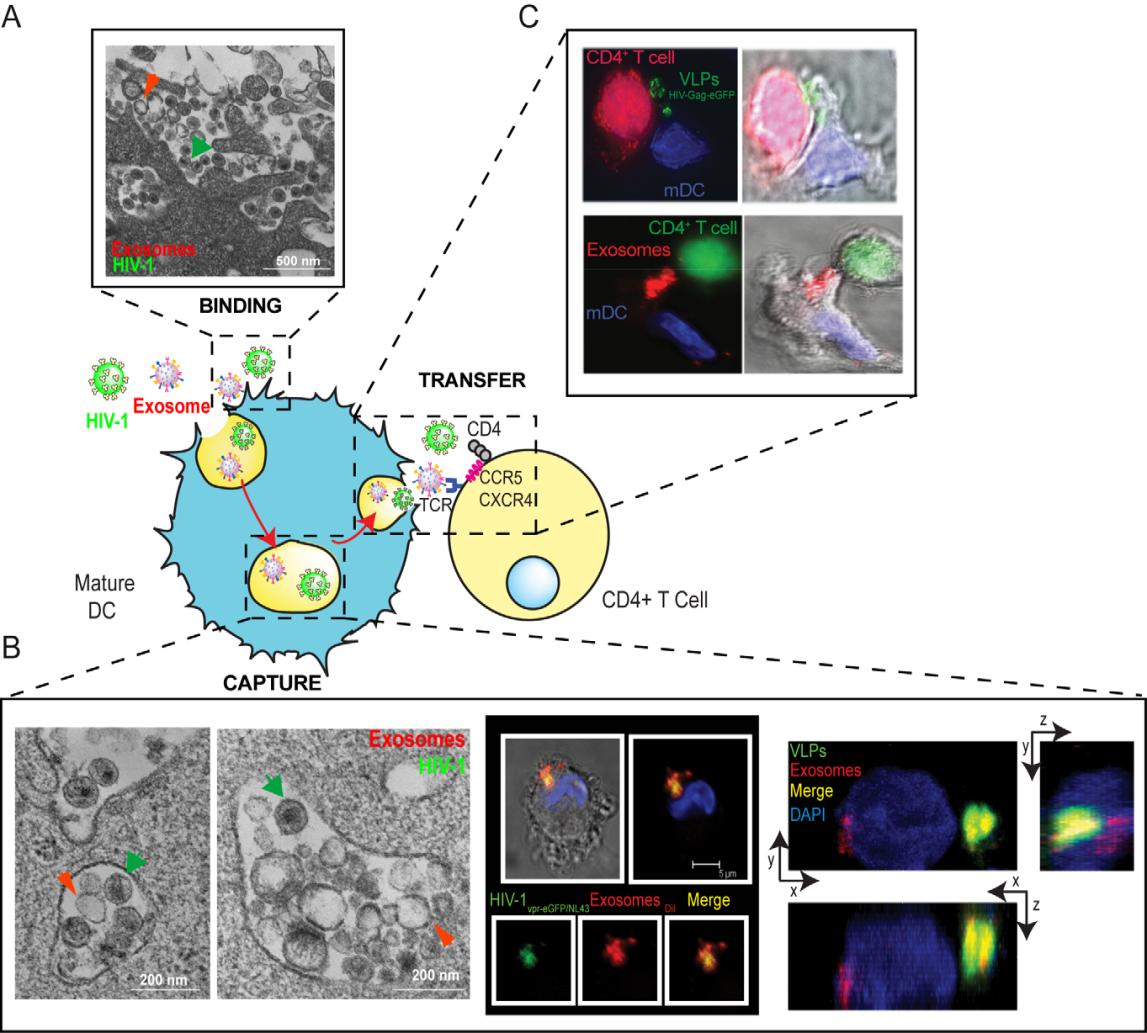
Capture and transfer of HIV-1 particles by mDCs converges with the exosome-dissemination pathway. (**A**) **Binding**. Electron microscopy images of mDCs simultaneously pulsed with HIV_NL43_ and Jurkat-derived exosomes. Particles displaying viral morphology (with an electro-dense core; green arrows) or exosome morphology (with lighter core; red arrows) accumulated in the same area of the membrane. (**B**) **Capture**. **Left**. Electron microscopy as in (**A**), showing HIV_NL43_ and Jurkat-derived exosome accumulation within the same vesicles. **Middle**. Confocal images of a section of an mDC exposed to HIV_vpr-eGFP/NL43_ and Jurkat-derived exosomes labeled with DiI for 4 h and stained with DAPI. Top images show the mDC, where the red and green fluorescence merged with DAPI either with or without the bright field cellular shape are presented. Bottom images show magnification of vesicles containing these particles where individual green and red fluorescence and the combination of both are depicted. **Right**. Confocal microscopy analysis of an mDC pulsed simultaneously with HIV Gag-eGFP VLPs and Jurkat-derived exosomes labeled with DiI and then stained with DAPI. Composition of a series of x-y sections of an mDC collected through part of the cell nucleus and projected onto a two-dimensional plane to show the x-z plane (bottom) and the y-z plane (right). (**C**) **Transfer**. Infectious-like synapses could also be observed in co-cultures where mDCs were previously pulsed either with HIV Gag-eGFP VLPs (**Top**) or Jurkat-derived exosomes labeled with DiI (**Bottom**), extensively washed, and then allowed to interact with Jurkat CD4^+^ T cells. Images shown, from left to right, depict the red and green fluorescence channels merged with DAPI, the bright field cellular shape, and the combination of both.

Upon maturation, DCs capture large amounts of HIV-1, HIV-Gag VLPs, and Jurkat-derived exosomes, accumulating these particles in the same intracellular compartment (Figure 4) that stains for tetraspanins such as CD81, is characteristic of multivesicular bodies, and is devoid of LAMP-1 lysosomic markers [63], as previously reported for HIV-1 [64],[65]. These data are in agreement with our previous findings regarding internalization of virus lacking envelope glycoprotein in mDCs and mature myeloid DCs [17].

Therefore, if exosomes use the same trafficking pathway in mDCs as HIV, the receptors dragged from the membrane of infected cells during viral budding that ultimately lead to viral capture should also be present in the membrane of exosomes. Interestingly, both exosomes and HIV can bud from particular cholesterol-enriched microdomains in the T cell plasma membrane [66]–[68], sharing glycosphingolipids and tetraspanin proteins previously used as bona fide lipid raft markers [64],[69],[70]. These similarities in composition and size are a strong argument for the Trojan exosome hypothesis, which suggests that retroviruses are, at their most fundamental level, exosomes [62].

We have further confirmed the existence of a common entry mechanism in mDCs by observing direct competition between different particles (HIV-1, HIV-Gag VLPs, MLV-Gag VLPs, and exosomes) derived from similar cholesterol-enriched membrane microdomains, which could not be inhibited by viral-size carboxylated beads or pronase-treated vesicular stomatitis virus particles budding from non-raft membrane locations [71]. Therefore, we consider that budding from lipid raft domains is essential to include specific mDC recognition determinants that allow viral and exosome capture [63].

Interestingly, a previous study revealed an association between endocytosed HIV-1 particles and intraluminal vesicle-containing compartments within iDCs [72]. However, the mechanism we propose differs from this previous paper in two fundamental aspects. First, the earlier work focuses on iDCs, and second, in their case, virus was endocytosed into the compartment where iDCs typically produce exosomes by reverse budding, thus contrasting with the gather mechanism of exosome and HIV uptake that we propose for mDCs. However, our findings concur because HIV-1 particles captured by iDCs were exocytosed in association with exosomes and could mediate *trans*-infection of CD4^+^ T cells [72]. Analogously, we found that mDC capture of HIV-1, VLPs, and exosomes allowed efficient transmission of captured particles to target T cells (Figure 4) [63].

## The Role of Glycosphingolipids during Capture

Our data also revealed that internalization of HIV-1, VLPs, or exosomes could not be abrogated with an effective protease pretreatment of either of these particles or mDCs [63]. Nevertheless, this observation does not exclude the potential role of proteins during viral or exosome capture, and might just reflect that the molecular determinants involved in capture were not fully processed by the proteases employed. However, treatment of virus-, VLP-, or exosome-producing cells with inhibitors of sphingolipid biosynthesis (such as fumonisin B1 and N-butyl-DNJ) extensively reduced particle entry into mDCs without interfering with their net release from producer cells. Although it has been previously shown that certain ceramide inhibitors diminish the infectivity of released HIV-1 particles after treatment of virus-producer cells [73] and can block HCV replication in vitro [74], at the viral input used in our study differences in infectivity were moderate. Moreover, treatment with a different agent that specifically blocks glycosphingolipid biosynthesis (N-butyl-DNJ) did not affect viral infectivity at all, while inhibiting viral mDC capture. Therefore, our findings establish a critical role for glycosphingolipids during mDC binding and endocytosis of particles derived from cholesterol-enriched domains such as HIV and exosomes [63],[75]. These data could imply a direct interaction of the glycosphingolipids with the plasma membrane of mDCs. Alternatively, the glycosphingolipids could maintain the structural entities required for viral and exosome binding to mDCs, allowing the interaction of pronase-resistant proteins with the mDC membrane surface. Further studies will help to clarify which of the two models our data support actually accounts for particle endocytosis.

## Concluding Remarks

The capture of retroviruses and exosomes is upregulated upon DC maturation, leading internalized particles into the same CD81^+^ intracellular compartment and allowing efficient transmission to CD4^+^T cells. This novel capture pathway, where retroviruses and exosomes converge, has clear implications for the design of effective HIV therapeutic vaccines. Although mDCs pulsed with inactivated virus could stimulate specific CD8^+^ T cell immune responses in infected patients, as reviewed in [76], these injected mDCs could also mediate *trans*-infection of new CD4^+^ T target cells, amplifying viral dissemination. Therefore, the safety of these strategies should be carefully evaluated, and preferentially explored in patients with undetectable viral load. Regarding prophylactic HIV vaccines, the proposed exosomal origin of retrovirus predicts that HIV poses an unsolvable paradox for adaptive immune responses [62]. Further work should address the specific differences between retroviral particles and exosomes to overcome these difficulties.

Taken as a whole, our results suggest that mDCs, which have a greater ability than iDCs to transmit the virus to target cells and interact continuously with CD4^+^ T cells at the lymph nodes—the key site of viral replication—could play a prominent role in augmenting viral dissemination. Underscoring the molecular determinants of this highly efficient viral capture process, where retroviruses mimic exosomes to evade the host immune system, could lead to new therapeutic strategies for infectious diseases caused by retroviruses, such as HIV-1, and T lymphotropic agents such as HTLV-1. Furthermore, this knowledge can help in the design of safer candidates for use in a DC-based vaccine.
